# What, Where, When and How of COVID-19 Patents Landscape: A Bibliometrics Review

**DOI:** 10.3389/fmed.2022.925369

**Published:** 2022-07-01

**Authors:** Kunmeng Liu, Xiaoming Zhang, Yuanjia Hu, Weijie Chen, Xiangjun Kong, Peifen Yao, Jinyu Cong, Huali Zuo, Jian Wang, Xiang Li, Benzheng Wei

**Affiliations:** ^1^Center for Medical Artificial Intelligence, Shandong University of Traditional Chinese Medicine, Qingdao, China; ^2^Qingdao Academy of Chinese Medical Sciences, Shandong University of Traditional Chinese Medicine, Qingdao, China; ^3^Department of Cardiovascular Surgery, The Affiliated Hospital of Qingdao University, Qingdao, China; ^4^State Key Laboratory of Quality Research in Chinese Medicine, Institute of Chinese Medical Sciences, University of Macau, Macao, Macao SAR, China; ^5^Warshel Institute for Computational Biology, The Chinese University of Hong Kong, Shenzhen, Hong Kong SAR, China; ^6^Science College, Shandong Jiaotong University, Jinan, China

**Keywords:** patent landscape, patent mining, bibliometric patent analysis, social network analysis, citation network, COVID-19, coronavirus

## Abstract

Two years after COVID-19 came into being, many technologies have been developed to bring highly promising bedside methods to help fight this epidemic disease. However, owing to viral mutation, how far the promise can be realized remains unclear. Patents might act as an additional source of information for informing research and policy and anticipating important future technology developments. A comprehensive study of 3741 COVID-19-related patents (3,543 patent families) worldwide was conducted using the Derwent Innovation database. Descriptive statistics and social network analysis were used in the patent landscape. The number of COVID-19 applications, especially those related to treatment and prevention, continued to rise, accompanied by increases in governmental and academic patent assignees. Although China dominated COVID-19 technologies, this position is worth discussing, especially in terms of the outstanding role of India and the US in the assignee collaboration network as well as the outstanding invention portfolio in Italy. Intellectual property barriers and racist treatment were reduced, as reflected by individual partnerships, transparent commercial licensing and diversified portfolios. Critical technological issues are personalized immunity, traditional Chinese medicine, epidemic prediction, artificial intelligence tools, and nucleic acid detection. Notable challenges include balancing commercial competition and humanitarian interests. The results provide a significant reference for decision-making by researchers, clinicians, policymakers, and investors with an interest in COVID-19 control.

## Introduction

Severe acute respiratory syndrome coronavirus 2 (SARS-CoV-2) is the virus responsible for the coronavirus disease of 2019 (COVID-19), which was first reported in Wuhan, China, in late December 2019 and then spread rapidly throughout world. SARS-CoV-2 belongs to the Betacoronavirus genus of the Coronaviridae family, and it primarily affects the respiratory system, although other organ systems are also involved. Symptoms can vary drastically; they include fever, chills, dry cough, sputum production, fatigue, lethargy, arthralgias, myalgias, headache, dyspnea, nausea, vomiting, anorexia, and diarrhea. Many countries and regions were blockaded due to transmission via droplets and aerosols (1, 2). To date, 532 million people have been infected, and 6.3 million people have died. The outbreak of COVID-19 has presented many challenges globally.

A large amount of academic literature on COVID-19 has been emerging in many disciplines, including a great deal of meta-research summarizing ongoing vaccine development or even drug discovery (3–5) and published papers (6–11). In addition, there is a rapidly emerging COVID-19 patent literature, despite the short time since the pandemic began. Patents have been discussed in the context of the pandemic but have received relatively little attention and analysis compared to the journal and preprint literature. Little attention has been paid to patent documents as a useful source of information.

A patent is considered an intangible asset that grants market exclusivity to an inventor for a new invention. Market exclusivity refers to enormous economic rewards available for an entity since it usually receives a monopoly for its invention over a crucial period of 20 years. Patented inventions represent technologies that the individuals or entities filing the patent consider to have commercial value. There is a strong positive relationship between science quality and patents, and the patent system has been regarded as a critical factor in promoting innovation in clinical medicine.

However, although one of the purposes of patents is disclosure, they are often vague and obfuscating, leaving little ability to scrutinize the underlying science (12). Stealth research is a term coined to describe “touted biomedical innovation happening outside the peer−reviewed literature,” i.e., outside scientific journal articles (13). Previous investigations of stealth research have focused on start-up companies or companies with high valuations (14). Owing to the damage caused by COVID-19, the Research and Development (R&D) section was vigorously embraced. Due to the awareness created among researchers, the development of unique technologies is currently associated with patenting inventions (15). As a result, an enormous number of patent documents have been registered by R&D in patent offices and are available in patent databases (16). The emerging COVID-19 patents provide an opportunity to explore this phenomenon in a different context.

Patent landscape analysis can be defined as an analysis of the relationships between multiple sets of indicators or those indicators measured against temporal, technical, or spatial dimensions (17). Social network analyses in patent landscapes can represent network connections or the density of clusters of scientific or technological fields (18). There is a lack of comprehensive bibliometric analysis on the patent landscape in the COVID-19 field (19–25).

To better understand the research dynamics during a pandemic, provide some evidence that patents might act as an additional source of information for mapping the COVID-19 literature and maximizing common interests by pooling COVID-19 patents, and fill the gap, we present a descriptive study and provide a scenario of the current R&D trends. This paper analyzes the global patent situation of COVID-19 to provide ideas for future research on the treatment of SARS-CoV-2.

## Materials and Methods

### Data Source and Search Strategy

Patent retrieval was conducted via the Derwent Innovation platform database on May 31, 2021, by establishing search terms related to “COVID-19” to search the titles, abstracts, and claims. The Guideline of Preferred Reporting Items for Systematic Reviews and Meta-Analyses was used as the reference to collect all patent documents worldwide and exclude unrelated data (Supplementary Figure 1). Two independent researchers (KM Liu and XM Zhang) screened the patents for eligibility. Any disagreements were resolved with reevaluation and consensus by researcher BZ Wei.

### Statistical Analysis

We conducted a bibliometric analysis to examine the current knowledge on technology-enabled COVID-19 patents. Unlike traditional reviews, a bibliometric review is a systematic analytical tool that helps researchers determine the most influential patents by employing a citation network analysis perspective (26). Networks are composed of 2 elements, nodes (patents or patent assignees) and edges (27). The edge directions of the citation network shift the research focus from a single patent to a citation relationship. The structure and characteristics of the patent citation network totally reflect the mode of technology flow with evolution. In addition, the cooperation network reflects the interaction between entities of technological creation. In addition, descriptive methods and text clustering were used to analyze the patent indicators.

### Analytical Tool

Cytoscape software was used to carry out visual analysis of the co-patent network of collaboration. Gephi software was used to make citation networks and create the milestones in the technology trajectory of patents. The force-directed layout algorithm was used in the network distribution. Microsoft Office Excel 2021 was used to process the data and analyze the time trends, patent ownership, organizations, countries, and technological categories. We used a variety of diagrams for visualization, such as bar charts, histograms, line charts, violin plots, themescapes of text clustering, heatmaps, sunburst charts, technology road maps, and network charts, to make more intuitive representations.

We reported items according to the Reporting Items for Patent Landscapes (RIPL) checklist (28, 29). The complete method of this study is shown in the Methods section of the Supplementary Material.

## Results

### Data Overview

**FIGURE 1:**
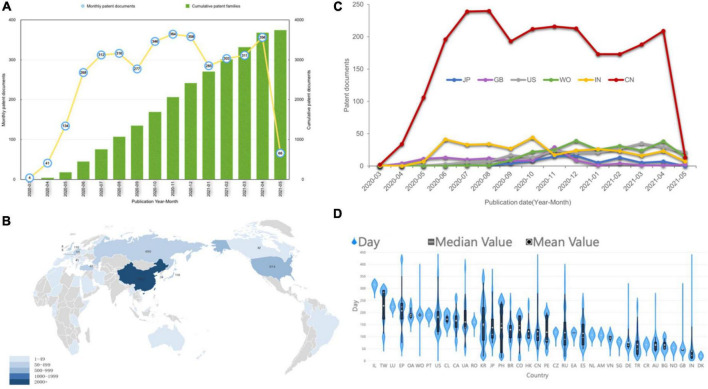
Current tendency for time and geographical distribution of COVID-19 patents. **(A)** The annual and cumulative number of COVID-19 patents. **(B)** The country landscape by nationalities of jurisdictions. The darker the color, the more patents. **(C)** The map shows the number of patent families by the top six locations of patent inventors temporally. **(D)** Violin plot of the time period between patent application and publication. The short white horizontal line is the median; the white dot is the mean; the black bar in the center of the violin is the interquartile range; and the black lines stretching from the bar are defined as the first quartile –1.5 IQR and third quartile + 1.5 IQR.

In total, the dataset has 3,741 patent documents with 3,543 DWPI patent families. In the 2 years since the outbreak of the epidemic, the number of COVID-19 patents has increased explosively, and the applications per month can number in the hundreds (Figure 1A).

In December 2019, there were three patent applications as the COVID-19 epidemic broke out. However, no patent was published after examination until March 2020. COVID-19 patents entered a period of rapid development after March 2020. Patent publications grew continuously until November 2020 and then entered a fluctuating growth mode. The number of patent applications reached its peak in March 2020 (Supplementary Figure 3A). There was an obvious lag between patent publication and application. In addition, after 2021, with the normalization of the epidemic situation, applications entered a weak stage (Supplementary Figure 3B).

### Geographical Distribution

Figure 1B shows the geographical distribution of COVID-19 patents by the nationalities of their inventors by highlighting the most productive countries, which include China (2,407 patents), the United States (974 patents), Russia (490 patents), Japan (118 patents), the United Kingdom (110 patents), Switzerland (88 patents), Turkey (67 patents), Germany (55 patents), etc. The two-letter codes are shown by full country name in Supplementary Table 2. The total ranking of different patent offices in which patents were filed is shown in Supplementary Figure 2. Patent applicants typically apply for patent protection in countries with potential markets of products because of regional restrictions. The patent jurisdiction refers to the region where the applicant wants the patent invention to be protected. In Figure 1C, COVID-19 patents are geographically distributed by the top five patent office’s annually. The United States and United Kingdom have increasingly become more important markets, while Germany and India have fallen into a relatively weakened status. China has always been in a monopoly position, but it was overtaken by the United States in May 2021. WO means the patent applications in Would Intellectual Property Organization by Patent Cooperation Treaty, and the increase in the number of patents filed by WO means that more attention was paid to the protection of the global market.

Denmark has the shortest average time for patent examination (Figure 1D). However, China, the United States and Japan, which are large patent holders, have a relatively long examination time. Patents of India and the United Kingdom have a short examination time.

### Patent Assignees

Among the main patent assignees, most come from academia and government, and the Chinese Academy of Medical Sciences appears in first place with 63 patent families (Table 1). The second-largest assignee is the PLA Academy of Military Medical Sciences, with 53 patent families. Regarding the top company assignees, Suntrap Life Technologies Ltd. holds the leading industrial position with 16 patents. The German company Bayer sits in 13th with an average number of patents per family of 1.57 and pays the most attention to patent portfolios.

**TABLE 1:** Leading COVID-19 patent assignees.

Rank	Assignee	DWPI families	Patent files	Average	Type
1	Chinese Academy of Medical Sciences	62	63	1.02	A&G
2	PLA Academy of Military Medical Sciences	53	53	1.00	A&G
3	Sun Yat-sen University	44	45	1.02	A&G
4	Fudan University	28	32	1.14	A&G
5	West China Hospital Sichuan University	27	28	1.04	A&G
6	Chongqing Medical University	27	27	1.00	A&G
7	Suntrap Life Technologies Ltd.	19	19	1.00	C
8	Jiangsu Provincial Center for Disease Control and Prevention	16	16	1.00	A&G
9	Zhejiang University	13	15	1.15	A&G
10	Tsinghua University	14	14	1.00	A&G
11	Lovely Professional University	13	14	1.08	A&G
12	Shanghai Jiao Tong University	13	14	1.08	A&G
13	Chinese Centre for Disease Control and Prevention	13	13	1.00	A&G
14	Peking University	13	13	1.00	A&G
15	Jilin University	10	13	1.30	A&G
16	Bioscience (Tianjin) Diagnostic Technology Co., Ltd.	12	12	1.00	C
17	Shanghai National Engineering Research Center for Nanotechnology Co., Ltd.	12	12	1.00	C
18	ACROBiosystems Co., Ltd.	11	11	1.00	C
19	Dalian Polytechnic University	11	11	1.00	A&G
20	Shanghai Public Health Clinical Centre	11	11	1.00	A&G
21	Rigel Pharmaceuticals	7	11	1.57	C

*C, commercial assignee; A&G, industrial and academic assignee; Average, Average number of patents per DWPI family.*

*Type: Institutional type of assignee.*

Supplementary Table 1 shows the patent family size in different countries. Italy trial with 1.57 average patent members, ranking the top. The small size of Chinese and Indian patent families, with only 1.04 and 1.02 members within a family on average, respectively, is worth noting, especially in comparison to other leading countries in COVID-19 patents. This kind of relatively inactive behavior on international patent applications provides additional evidence of the lack of an international strategy among Chinese and Indian applicants.

**FIGURE 2:**
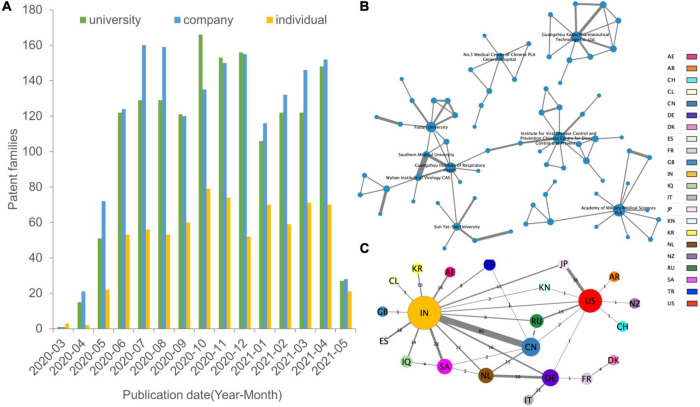
Spatial and institutional dimensions of research collaboration. **(A)** The bar chart shows the patent numbers for each assignee type of institution. **(B)** Top collaboration partners. The nodes (organizational patent assignee colored with country) and edges (collaboration) in the network visualization map represent the co-assignee relations. The institutional collaboration network does not include the individual assignee and labels names of top active institutions by Cytoscape. **(C)** Networks of country relationship modes. Nodes (individual and organizational patent assignees) denote locations, and edges denote the count of domestic, bilateral, and multilateral country collaborations. Node size is scaled to the numerical value of the network degree (co-patent number), while the thickness of the edges is determined by the numerical value of the network weighted degree (frequency of cooperation). Countries are represented in different colors. The regional cooperation network is displayed in the “yFiles Organic layout” based on the force-directed layout paradigm by Cytoscape.

Figure 2A shows the distribution of different types of assignees temporally. The company is the type of assignee with the most (39.36%) patents first deposited, followed by academia and government and individual. Patent applications by companies and universities maintained a relatively stable growth trend. Universities exceeded companies in late 2020. However, all kinds of patents received showed a sharp decrease after May 2021.

There are 74 nodes (assignees) and 96 edges (coownership relationships among assignees) in Figure 2B, which are the main collaborative patterns and relationships among institutional collaboration assignees. To achieve better visual performance, the network only shows the clusters containing more than six organization members and gives the label name of the most active partners. Supplementary Figure 7 shows the information-rich institutional cooperation network. The Institute for Viral Disease Control and Prevention of the Chinese Centre for Disease Control and Prevention, PLA Academy of Military Medical Sciences, Guangzhou Institute of Respiratory Health, Guangzhou Kaipu Pharmaceutical Technology Co., Ltd., and Fudan University have active cooperative relationships, forming an extensive collaboration network. Southern Medical University and Wuhan Institute of Virology have the closest collaboration.

To characterize regional collaboration, the collaboration network was transformed into a regional network (Figure 2C). Nodes of the network denote regions, and edges denote collaborative relationships between co-assignees. The cross-regional network includes 22 nodes and 33 edges overall. India is dominant in developing broad partnerships with different countries. At the same time, India and China are partners with the highest frequency of cooperation, reaching 80 times, far higher than other partners. In contrast, although China is the third-largest node after India and the United States, it has relatively few partners and cooperation with its neighbors. European countries are connected in parallel with the central nodes of India and the United States in the network, with the only exceptions being Germany linking to 5 neighbors, suggesting that it lacks a centralized system for developing COVID-19 technologies.

### Technological Characteristics

**FIGURE 3:**
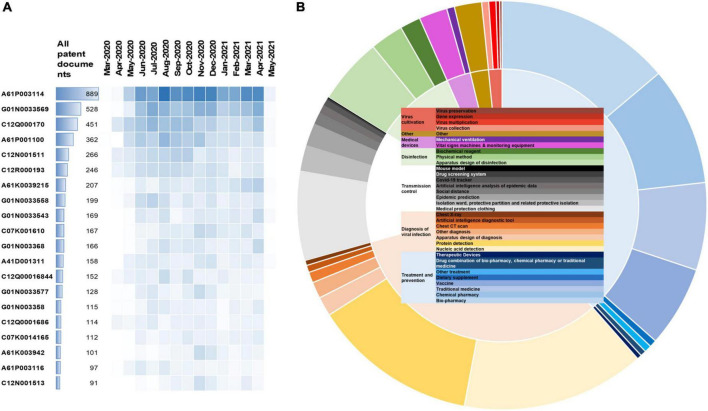
Distribution of the types of functional classification of COVID-19 patents. **(A)** The temporal evolution of the leading 20 International Patent Classification (IPC) codes. **(B)** Sunburst chart by focused functions. The area of the graph represents the percentage of technology.

Figure 3A shows that, in the early 2000s, the two areas with the most patent applications were A61P003114 (anti-infectives for RNA viruses and G01N0033569 for investigating or analyzing materials for microorganisms, for further information on IPC codes, visit^1^ and Supplementary Table 3). By the end of 2020, hot spots of C07K001610 (immunoglobulins from RNA viruses, e.g., monoclonal or polyclonal antibodies) and G01N0033577 (investigating or analyzing materials by specific methods involving monoclonal antibodies) emerged. After 2021, the new trends were A61K0039215 (medicinal preparations containing antigens or antibodies for coronaviridae) and G01N003368 (investigating or analyzing materials by specific methods not covered by groups involving proteins, peptides or amino acids).

To refine this patenting landscape from a technical point of view, the patents were sorted manually, and a sunburst diagram was generated, which shows 33 subcategories (Figure 3B). All patents were classified into 7 groups of technology uses, with “treatment and prevention” (38.48%) and “diagnosis of viral infection” (32.19%) constituting the two most frequently claimed uses. Of the subgroups of technologies, “Nucleic acid detection” was given priority (14.48%). Accounting for 13.83% of all the patents retrieved, the “Biopharmacy” area was the second-largest subgroup of technologies.

To understand how the different applications have changed over time in terms of the direction of technology and investment trends, we analyzed the annual change (Supplementary Figure 4). “Vaccine,” “Social distancing,” “Epidemic prediction,” “Artificial intelligence diagnostic tool,” “Artificial intelligence of epidemic data,” and “Traditional medicine” showed a constant growth from mid-2020 to early 2021.

A geographic breakdown showed the distribution of patents in the technology fields across different jurisdictions. China not only covers all technologies but also leads in almost all top technology fields, except medical devices. Assignees in China focus more on the diagnosis of viral infection, while Indian assignees focus more on “transmission control,” and the United States focus more on “treatment and prevention.” For subfields, China not only has been a pioneer in “Nucleic acid detection,” with 456 patents compiled in this subcategory, but is also a leader in “Protein detection” (Supplementary Figure 5). “Medical protection clothing” is the largest depositor in India. Would Intellectual Property Organization and United States focus, respectively on “Biopharmacy” and “Chemical pharmacy.” The inventions classified as “Chemical pharmacy” were the richest in United Kingdom applications, while Japan hits mostly on “Medical protection clothing.” In addition, the patent landscape shows fields with high patent (dot) activity labeled by the peaks “Chinese traditional medicine,” “Primer Kit Sequence,” and “disease anti-inflammatory syndrome” (Supplementary Figure 6).

### Milestone Patents

**FIGURE 4:**
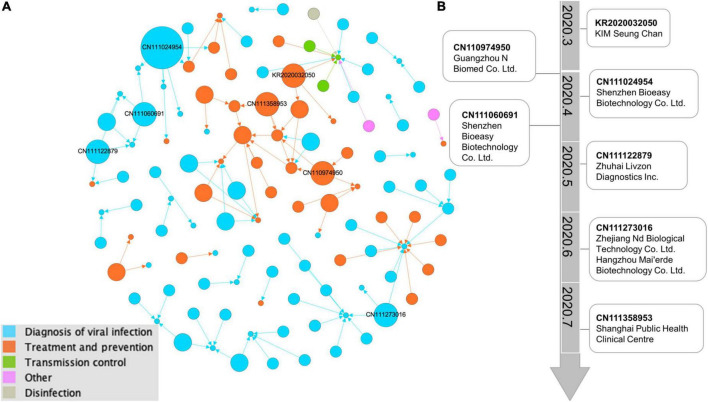
Network visualization map of citations by COVID-19 patents. **(A)** Citation network illustrating the evidence sources cited in 117 recommendation patent documents. The larger the circle is, the more frequently the patents received citations. Lines represent the citation direction. The force-directed layout algorithm of “Fruchterman Reingold” by Gephi is used to make the network distribution. Different circle colors indicate different technologies. **(B)** The main path from the typical key-route citation weighted with the top 10 patents of citation out-degree. In the visual analysis of the main path based on patent citation, time was added to the technology trajectory calculated by Gephi software, which clearly shows the research focus and direction of this field in different time periods.

Based on the technical investigation, we constructed a global patent citation network that has 117 nodes and 113 edges (Figure 4A). We set the node size according to the out-degree, that is, the larger the out-degree, the larger the node size, and the more references a given patent received. Nodes of different colors represent different technologies. Most of the top-cited patents are located in the blue clusters. Apparently, most of the linked nodes are of the same color. The patent types “Diagnosis of viral infection” and “Treatment and prevention” form the main part of the citation network.

The backward citation data from all the COVID-19 patents were used to map the technological route by milestone patents to identify the emerging technologies among the 3,741 patents (Figure 4B). The milestone patents with relevant assignees were highlighted, thereby showing the most important assignees from China. There are three trends by information dissemination of the routes in COVID-19 inventions: drugs, vaccines and detection. The oldest patent KR2020032050 at the top is performing a COVID-19 suitable triple knockout DNA remedy that involves targeting coronaviruses by using complementary single-strand DNA oligomers. CN110974950 deals with technologies describing the vaccine composition that is useful for preventing SARS-CoV-2 infection. The highly cited patent CN111024954 describes the colloidal gold immunochromatography device useful for joint detection of COVID-19 antigens and antibodies. CN111060691 is related to a fluorescence immunochromatography device useful for detecting the novel coronavirus COVID-19 and comprises test strips. As the route continues to more recent patents, CN111122879 is related to coating solutions useful in the preparation of coronavirus antibody detection products. CN111273016 describes a kit based on S protein–ligand and ACE2 receptor competition method chromatography fast detecting coronavirus. CN111358953 develops a new vaccine vector that is useful for efficiently inducing the humoral immune response in the human body.

## Discussion

COVID-19, a novel pandemic disease, has been a global problem since December 2019. Patent documents are a potentially useful resource, but they are often underutilized. Characterizing the patent landscape of COVID-19 may be useful for informing research and policy. This study presents a patent landscape of COVID-19, including the temporal trend, organizational patentees, co-assignee scope, geographic activities, and technological focuses.

### Temporal

It has been 2 years since the coronavirus turned the world upside down. The epidemic broke out in December 2019, patent application began in December 2019 (US10722506 and US20200101060, for “Chemical pharmacy”), and the first patent disclosure occurred 3 months later (CN110870402 for “Traditional medicine”). The World Health Organization announced that the COVID-19 outbreak was a public health emergency in January 2020 and changed it to a pandemic event in March. The virus has infected almost two hundred and eighty million people and killed over 5.4 million (30). COVID-19 developed rapidly, which is consistent with the explosive growth of COVID-19 patents. The growth and fluctuation of the number of patents are roughly similar to the fluctuation trend of the number of epidemic infections (Supplementary Figure 8). COVID-19 ushered in a climax in winter, accompanied by a sharp increase in the number of patents.

### Technical

COVID-19 patients have different conditions, symptoms and severity. Scientists are committed to finding appropriate methods for the pandemic to treat and control its spread. Researchers from all disciplines come together to provide expertise (6–11) as the COVID-19 pandemic unfolds. Viral ribonucleic acids can be detected by real-time polymerase chain reaction. Great efforts are being made to develop a precise and rapid diagnostic tool based on CRISPR/Cas13. Preventive measures such as social distancing, hygiene maintenance, and contact tracing are approaches used to manage the pandemic. Experimental vaccines and therapeutic drugs to prevent and treat COVID-19 are emerging at an unprecedented rate. Because of the unprecedented nature and urgency of this health challenge, many interventions, from the use of off-label drugs, stem cell therapy, and drug repurposing to convalescent patient serum, have been were mobilized (31–33). Additionally, non-toxic and innovative therapeutic agents are being explored. There are many noteworthy manifestations in patents.

For example, traditional Chinese medicine (TCM) has proven to be effective in the treatment of COVID-19 by reducing patients’ symptoms and adjusting their immune systems (34). Since the epidemic, the National Administration of Traditional Chinese Medicine has held online events introducing TCM as a method of treating COVID-19 (35). TCM products were sent to countries and regions worldwide in need, and TCM experts have traveled to many countries to guide local epidemic work, which led to a large number of related patents. In addition to TCM herbs, acumoxa therapy (WO2020245804) and moxibustion treatment (CN112043771) are bright spots.

Furthermore, in the case of severe virus mutation, personalized immunity has received attention. The Omicron variant is 5.4 times greater than the Delta variant. Omicron is four times more infectious than the Alpha strain and twice as infectious as the Delta strain (36). Tevogen Bio issued Patent US11191827, which significantly reinforced Tevogen’s intellectual property position in the use of SARS-CoV-2-specific cytotoxic CD8 + T lymphocyte immunotherapy for the treatment of COVID-19. The therapeutic targets are not affected in the Delta variant and appear to be preserved in the Omicron variant. Given some hurdles remaining in epidemic control, cutting-edge technologies such as monoclonal antibody therapy (US10787501), CRISPR-Cas-based assays in medical treatment and diagnosis (WO2021011504, CN111996236), and gene therapy tools (CN112138152) have been emerging. Thus, despite the lengthy time spent on the R&D of COVID-19 treatment technologies, the hope is truly coming to realize their therapeutic promise (37, 38). Affordable personalized immunotherapies are the next frontier of medicine, and disruptive business models are required to sustain medical innovation in the post-pandemic world (39). At the same time, “New Uses for Old Medications” have been developed for the treatment of COVID-19, such as azithromycin (US20210015837), baricitinib (IN202131015870), and favipiravir (CN111557939).

On the one hand, in the current world scenario due to COVID-19, people not only need to increase the immunity of their physical health but also should consider immunity to their mental health. Many people, such as doctors, nurses, policemen, and all frontline workers, will have their own stress levels on their work (40). Psychotherapy patents were born (IN202041043167, US20200327977). In addition to timely interventions, on the other hand, to solve the pandemic issue, research on predicting the disease (CN112185560) is extremely vital for buying more time for clinical trials and conducive to the intervention and prevention of COVID-19 by departments at all levels (41, 42).

Additionally, since the outbreak of coronavirus disease, with the increase in virus diagnosis, diagnostic efficiency has improved. This is of great significance for early isolation, disease treatment and the control of transmission. Compared with protein detection, nucleic acid detection is more sensitive and is the “gold standard” of laboratory detection (43). Therefore, the number of patients with nucleic acid detection is the largest. At the same time, AI-assisted computing has greatly improved the efficiency of new drug research and development (WO2021003196), diagnosis (CN111639676), and disease prediction (CN112582074). Attention should be paid to ramping up investment in artificial intelligence and facilitating the compulsory licensing of patents and transfer of know-how (44).

China’s COVID-19 strategy has been to identify and interrupt community transmission through swift containment measures, sometimes for whole cities, even though 75% of the population has already been vaccinated (45). Therefore, social distancing, detection, epidemic prediction, and vaccine patents in China have been promoted. “Adjusting to the new normal” is a phrase that can be used to describe the world’s response to the events of 2021. Masks have become the norm, physical distancing is a must, vaccines have been mandated, parts of the world are returning to lockdowns, and developing drugs and vaccines is urgent. These tactics have led to many related patents. Almost 2 years into the pandemic, it is clear that COVID-19 will be around for the near future, and we all have to adjust to it (46).

### Spatial

The number of Chinese patents is far ahead in terms of creativity and protection because of early experience and policy attention. China is a country in which COVID-19 is being discovered. There is a prioritized examination pilot in China and the US (47). In China, COVID-19 vaccine patents enjoy prioritized examination, such as the first vaccine patent CN111218459. The USPTO has implemented the COVID-19 Prioritized Examination Pilot Program to prioritize the examination of certain patent applications for COVID–19 use. At present, the US and India rank first and second, respectively, in coronavirus cases worldwide. Therefore, the number of patents in the two countries is also at the top of the list. Russia is the second-largest epidemic-infected country in Europe, which has caused the country to vigorously invent relevant patents. Italy and other European countries have shown advantages in patent portfolio layout, since it has a patent box (48), which is a low-corporate-tax regime used by several countries to incentivize research and development by taxing patent revenues differently from other commercial revenues.

### Organizational

As the top assignee, there is a military role of the PLA Academy of Military Medical Sciences in medical research. The Wuhan Institute of Virology announced that it applied for a patent for Gilead’s Remdesivir for treating COVID-19. However, the China National Intellectual Property Administration published the application CN11126553 entitled “Use of substituted aminopropionate compounds in the treatment of 2019-nCoV infection.” The role of the military in COVID-19 should not be underestimated.

Commercial competition has also begun. As the top assignee in industry, China’s Suntrap Life Technologies Co., Ltd., has taken the lead in establishing the Suntrap International Drug Discovery Network Alliance platform to serve as an international drug discovery network alliance. Furthermore, Gilead will not charge royalties for the sale of remdesivir drugs (WO2019014247, WO2017049060) in low-income countries. As long as COVID-19 is still listed as a public health emergency of international concern by the WHO, it will be exempted from royalties for sale of generic drugs covered by non-exclusive voluntary licenses agreements (49).

Ultimately, in the licensing of life-saving medicine, Merck does not prioritize public health. As a case in point, since the outbreak of the pandemic, Emory University, one of the top three American universities to receive COVID-19 project support through public sources, invented the antiviral pill entitled Molnupiravir (WO2021159044) with licensing rights owned by Merck. The Medicines Patent Pool (MPP) is a voluntary licensing and patent pooling. There is “a termination for challenge” clause in the agreement between Merck and the MPP of authorizing generic manufacturers from a limited number of low- and middle-income countries. This could undermine the production of cheaper generic versions of Molnupiravir.

Meanwhile, Pfizer’s recent license with the MPP for the drug ritonavir (Paxlovid) similarly excludes middle-income countries. In addition, Moderna has filed patents (US9364435, US8058069) isolating a gene sequence used in a vaccine to trigger an immune response to coronavirus. Only Moderna scientists were listed as inventors, although Moderna described the National Institute of Health of U.S. as “collaborators” (50). Clearly, a pandemic should not be a race between companies but the competition between humans and viruses. Countries and companies worldwide ought to do their best to cooperate with each other to make epidemic emergency situations easy to track.

### Social

The licensing deal struck was established among COVID-19 Technology Access Pool (C-TAP), MPP and Spanish National Research Council for a serological antibody technology regarding the screening for anti-SARS-CoV-2 antibodies (51). Health-driven, voluntary, non-exclusive, transparent, and open licenses were supplied for sharing data and information on COVID-19 treatment, diagnosis, vaccines, and other cutting-edge health technologies. Pioneers of COVID-19 life-saving tools can boost manufacturing ability and extend access to health products in all regions (52).

Unavoidably, free patents weaken innovation, and balance is also essential. Patent buyouts constitute a sound opportunity to provide access to the most effective vaccines to all countries during this severe period of the pandemic (53). Unlike patent waivers, there is no need in buyouts to impair private incentives for further innovation (54). Buyouts stimulate vaccines against mutations, produce sustainable population immunity at high levels and boost resistance to viral mutations. As opposed to waivers and compulsory licensing, buyouts incentivize the voluntary licensing of technical and business management knowledge (55). There is a high valuation of patented technology in patent licensing of such buyout auctions (56). Patent buyouts picked by the Covid-19 Vaccines Global Access initiative have the potential to facilitate global production of vaccines, change the monopoly pricing of private vaccines worldwide, and reduce prices to lower manufacturing costs in poorer countries (57).

The purpose of patents is to balance innovation with common interests (58); however, in a pandemic, common interests should take precedence. The importance of this work lies not only in preserving critical research but also in modeling a pragmatic mechanism for facing and solving the crisis. Nevertheless, recent commercial competition has raised serious concerns about access to healthcare and equitable global distribution (59).

During the pandemic, more robust teamwork between industry and society is needed, along with a reduction in intellectual property barriers (60), as exemplified by the public–private partnership between Bayer and Berkeley. Specifically, the city of Berkeley has established a drive-through COVID-19 vaccination clinic to support the mRNA vaccine of CureVac of Bayer. The imperative for solutions demands rethinking traditional manufacturing paradigms to engender wider access, bringing specialized medicines to specific patient populations and fostering in-depth cooperation. Clearly, a global pandemic is an all-hands-on-deck circumstance, a clarion call for greater collaboration.

In the future, collaborative efforts may increasingly harness the power of global innovation to meet the COVID-19 challenge. Cell and gene therapy, for instance, is still in its infancy. To date, no consensus platform exists for monoclonals. Meanwhile, global teams collaborate with internal and external innovation engines, leveraging expertise in chemistry, manufacturing, control strategies, and process engineering to ultimately deliver these solutions to patients on a global scale.

### Limitations of the Current Study

Notably, this study has some limitations. Not all inventions meet the criteria for patentability, and inventors may rely on other appropriate means of secrecy to protect their inventions. Although strict search criteria are established to retrieve patents related to COVID-19 as comprehensively as possible, they also limit the absolute scope of patent searches. Additionally, there is usually a time interval of at least 18 months between the patent application and publication, so the unpublished patent cannot be retrieved. These are, however, a general limitation of all patent landscape analyses.

## Conclusion

COVID-19 treatment and prevention technologies hold immense promise, and the number of patent applications is growing rapidly. To defeat the pandemic, an open innovation model highlighting academic-industrial partnerships has already been established. Currently, China is in a leading position, holding the greatest number of patents, which cover all relevant technologies and possess the majority of the top assignees, and India is most widely linked to other countries. In recent times, personalized immunity, TCM, epidemic prediction, and artificial intelligence have become research priorities. Given the collective threat of an epidemic, patent rights cannot remain mired in a pre-COVID conception of normalcy; greater efforts must be made to balance commercial interests with humanistic urgency. It is necessary to reinforce a “public-oriented” global project to ensure universal access to COVID-19 treatment and prevention. Faced with worldwide threats of infectious disease, major countries are duty-bound to rally leading public organizations and industry to respond to calls from developing countries. As diverse potential resources and geopolitical forces are currently available, regulatory forces should be responsive to the imperative for the rapid drafting of such innovative domains.

## Data Availability Statement

All data included in this study are available upon request by contact with the corresponding author.

## Author Contributions

All authors listed have made a substantial, direct, and intellectual contribution to the work, and approved it for publication.

## Conflict of Interest

The authors declare that the research was conducted in the absence of any commercial or financial relationships that could be construed as a potential conflict of interest.

## Publisher’s Note

All claims expressed in this article are solely those of the authors and do not necessarily represent those of their affiliated organizations, or those of the publisher, the editors and the reviewers. Any product that may be evaluated in this article, or claim that may be made by its manufacturer, is not guaranteed or endorsed by the publisher.

## Footnotes

1
https://www.wipo.int/classifications/ipc/en/
