# Supplementary material for: What, Where, When and How of COVID-19 Patents Landscape: A Bibliometrics Review
Source: Front Med (Lausanne). 2022 Jul 1;9:925369. doi: 10.3389/fmed.2022.925369 (PMC9283760; doi:10.3389/fmed.2022.925369)
Supplement: Supplementary file 1 [file Data_Sheet_1.docx]

Supplementary Material

**1. METHODS**

**1.1 Patent Search**

The Derwent Innovation® (Clarivate Analytics®) platform(1) was used for patent data-mining to achieve a dataset of patents dealing with inventions related to COVID-19 technologies. We collected the patent detailed information based on the DWPI patent family, which included the basic patent filed in the original country and subsequent equivalent patents on the same invention filed in different countries or offices. A search query was developed to retrieve patents related to COVID-19 invention which were mentioned in the Title (original and DWPI), Abstract (original and DWPI), Claims (original) of patents. The following search query were used:

Search Query:

CTB=("SARS-CoV-2" or "Severe acute respiratory syndrome coronavirus 2" or "COVID-19" or "coronavirus disease 2019" or "nCoV-2019" or "2019-nCoV" or "new corona virus*" or "novel corona virus*" or "new coronavirus*" or "novel coronavirus*" or "human coronavirus 2019" or "HCoV-19") OR ABD=("SARS-CoV-2" or "Severe acute respiratory syndrome coronavirus 2" or "COVID-19" or "coronavirus disease 2019" or "nCoV-2019" or "2019-nCoV" or "new corona virus*" or "novel corona virus*" or "new coronavirus*" or "novel coronavirus*" or "human coronavirus 2019" or "HCoV-19") or TID=("SARS-CoV-2" or "Severe acute respiratory syndrome coronavirus 2" or "COVID-19" or "coronavirus disease 2019" or "nCoV-2019" or "2019-nCoV" or "new corona virus*" or "novel corona virus*" or "new coronavirus*" or "novel coronavirus*" or "human coronavirus 2019" or "HCoV-19"); The search was performed at May 30th, 2021.

**1.2 Dataset cleaning**

A fundamental step of data normalization was performed to clean duplicate assignees and inventor’s names due to abbreviations, misspelling, initialization and international variation in the document records. The unrelated patents were deleted in the document records after being double-checked manually (Supplementary Material Figure 1) Moreover, after judging the relevance of technologies to patent files by a hierarchical reading order(2) from title, abstract, claims and full text, we added technology labels to relevant patent records.

**1.3 Cooperation Network**

In the study, for all institutional or regional collaboration networks, we used the Cytoscape (https://cytoscape.org/) software to visualize network. Cytoscape provides the basic functionality of layout and query networking and can be used to implement our data visualization. The strategy of “yFiles Organic layout” (2), which is a multi-purpose layout style based on the force-directed layout paradigm, was used as the network layout. In the network, the nodes represented the assignees, and the edges denoted the co-assignee relationship. The size of the node represented the patent family count, and the thickness of the edge indicated co-assignees’ count. The nodes were colored based on the country of assignees and distributed according to the “yFiles Organic Layout” strategy, with which nodes with more patents and stronger interconnectedness were placed in a more centered position. All institutions with external collaborations were displayed. The clusters with more than 6 nodes were thus extracted to generate isolated largest cooperative group.

**1.4 Landscape and identification of emerging fields**

We used large-scale text and data processing analysis based on text clustering and a visual map provided by Derwent Innovation tool named Themescape^TM^(3). Both are automated software which cluster similar terminology found in the titles, claims and abstracts of the dataset of 3741 patent documents related to COVID-19. The text clustering tool automatically grouped textual information within these fields into clusters based on their content similarity and it was used to discover meaningfully implicit subjects throughout all the patent documents. The results of the text clustering list showed more than one word separated by commas which represented a related broader subject. The patent landscape by ThemeScape™ provided an outcome in a map format where dots represented patents, and those with similar terminology were in close proximity due to sharing more phraseology than those located apart.

**1.5 Citation network and identification of emerging technologies**

Information based on patent citations from a group of patents can be used to forecast emerging technologies in a determined technological area(4, 5). Thus, we created a citation network to uncover the clusters of technological trends in COVID-19 inventions based on knowledge flows by mapping the main path of patent citation. Following a similar approach for the assembly of the cooperation network, the citation one was created using information from the “Publication number” (source) and “Cited refs” (target) columns from the original dataset. Gephi(https://gephi.org/) software was used to visualize citation network layout. The dataset containing the link between the citing-cited patent were imported into Gephi to generate the global patent citation networks. The network of patent citation was a directed graph, which represented the link from the citing patent to the cited patent. The global network of patent citation related to COVID-19 was designed by 117 nodes and 113 edges. The node size was set according to its out-degree value, which in this case related to the number of citations a patent received, that is, the greater the out-degree, the larger node size, and the more citations a given patent received. The nodes were colored based on different technology. The nodes were distributed using the “Fruchterman Reingold” (6) following manual adjustment, which was a force-directed layout algorithm.

**1.6 Classification of patents**

We collected the information about the classification of patents of technological characteristics based on previous research in order to capture technological features of the patent files. According to the previous literature and the actual situation of patent application, the overall patents were classified into 7 general categories and 33 subcategories. We further marked classification labels by a hierarchical reading order from the title, abstract, claims, and full-text. Supplementary Material Table 4 shows more details.

**2. SUPPLEMENTARY MATERIAL REFERENCES**

1. Reuters T. Derwent Innovation Indexsm Patent Database (2014). Available from: <https://www.derwentinnovation.com>.

2. Lyu L, Feng Y, Chen X, Hu Y. The Global Chimeric Antigen Receptor T (Car-T) Cell Therapy Patent Landscape. *Nat Biotechnol* (2020) 38(12):1387-94.

3. Analytics C. Derwent Innovation: Explanation on Themescape (2018). Available from: <https://support.clarivate.com/Patents/s/article/Derwent-Innovation-Explanation-on-ThemeScape?language=en_US>.

4. Picanço-Castro V, Pereira C, Covas D, Porto G, Athanassiadou A, Figueiredo M. Emerging Patent Landscape for Non-Viral Vectors Used for Gene Therapy. *Nat Biotechnol* (2020) 38(2):151. doi: <https://doi.org/10.1038/s41587-019-0402-x>.

5. Pereira CG, Picanco-Castro V, Covas DT, Porto GS. Patent Mining and Landscaping of Emerging Recombinant Factor Viii through Network Analysis. *Nat Biotechnol* (2018) 36(7):585-90.

6. Fruchterman TMJ, Reingold EM. Graph Drawing by Force-Directed Placement. (1991) 21(11):1129-64. doi: <https://doi.org/10.1002/spe.4380211102>.

**3. SUPPLEMENTARY MATERIAL FIGUREURES**


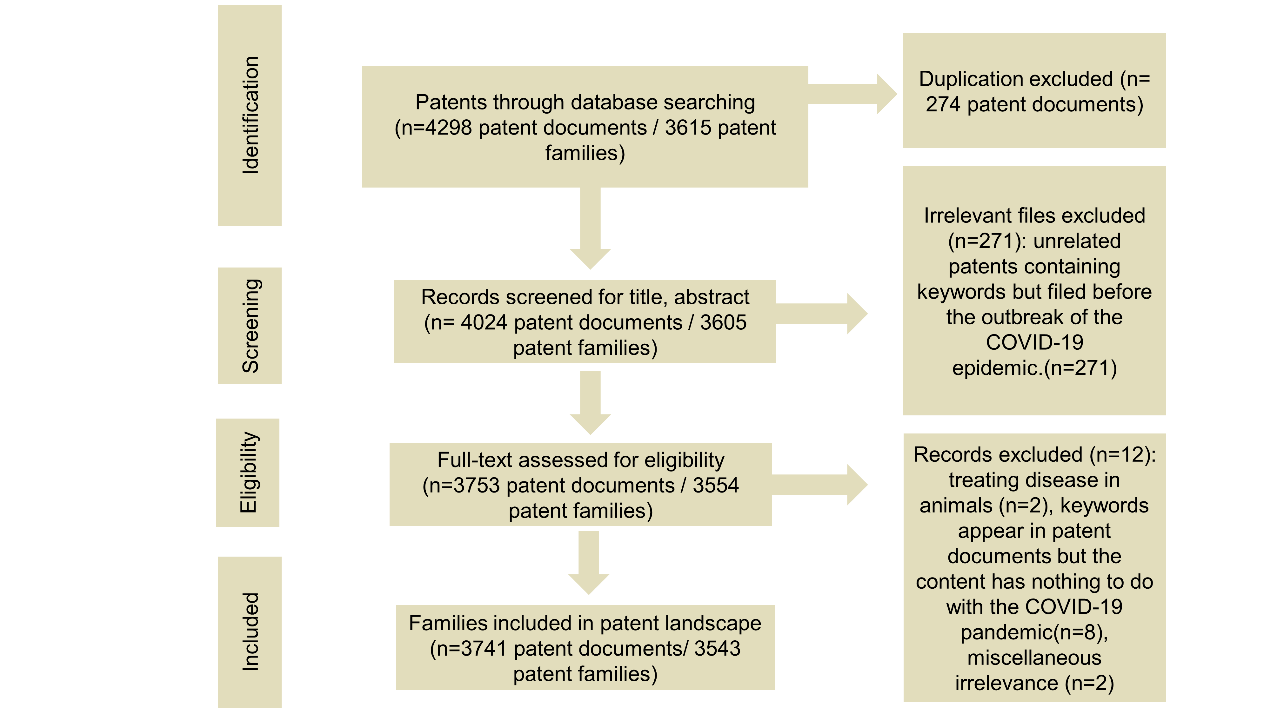


**Figure.1 PRISMA flow diagram detailing the number of patent documents included at each stage and the reasons for removal.**


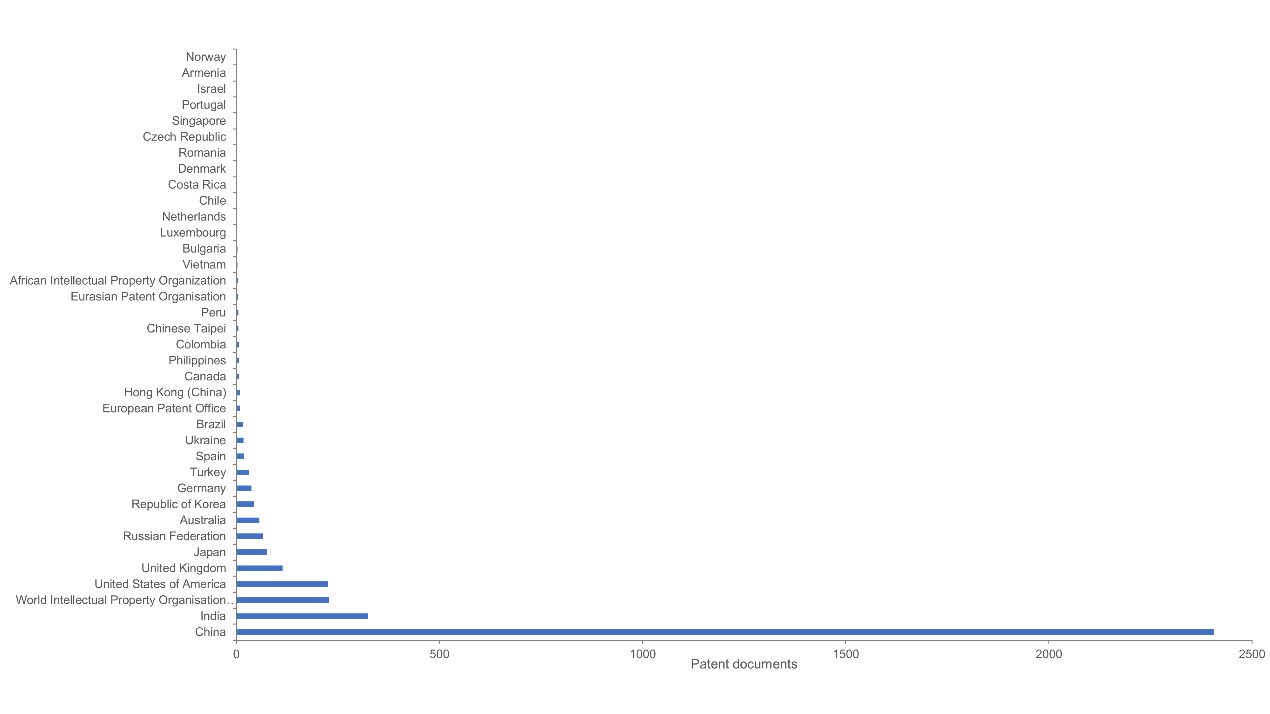


**Figure.2 The cumulative number of COVID-19 patents by jurisdiction filing patents.**


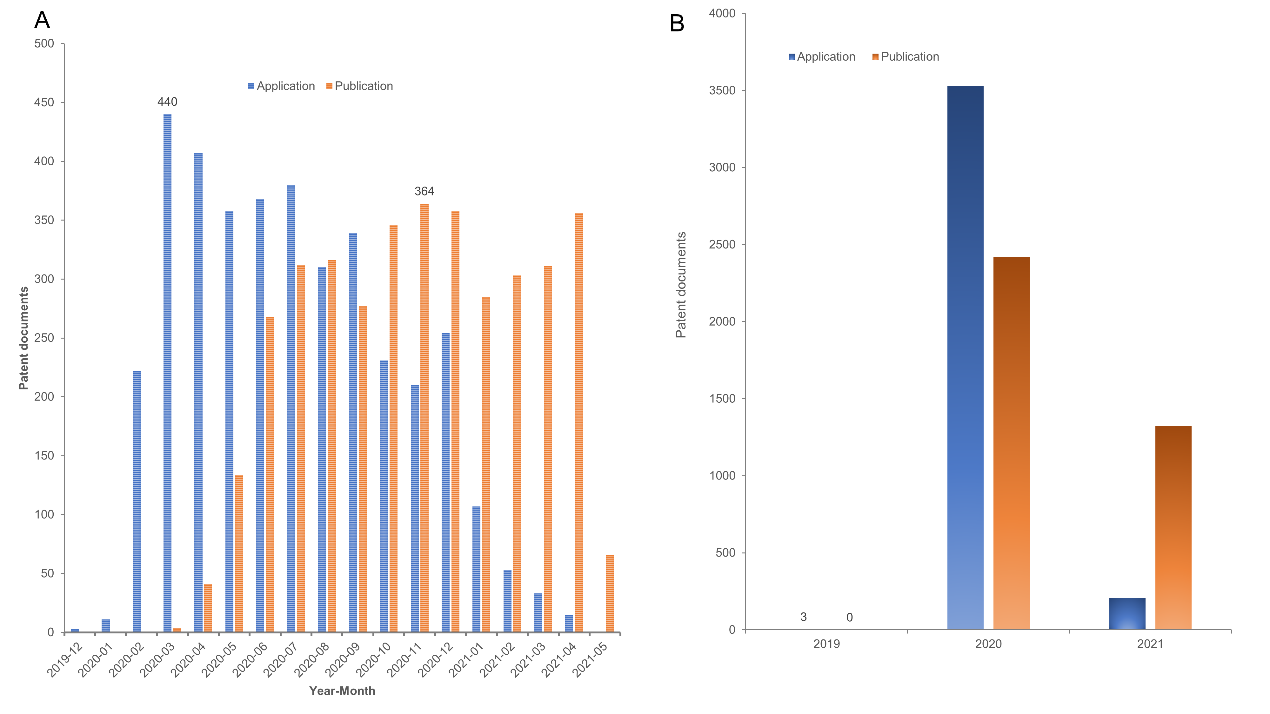


**Figure.3 Publication and application trend of COVID-19 patents.**

**(A). Distribution by month. (B). Distribution by year.**


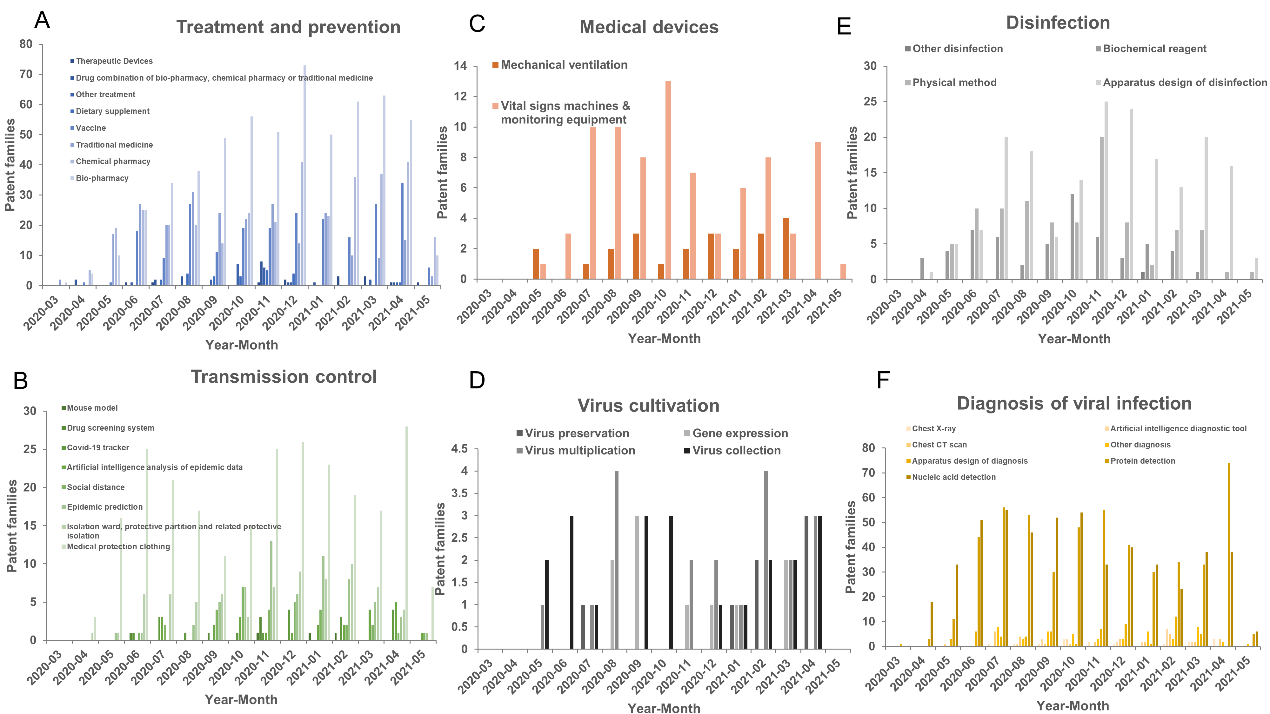


**Figure.4 Number of patent families related to the COVID-19 per technical category by time. (A)(B)(C)(D)represent different technology types in groups and subcategories.**


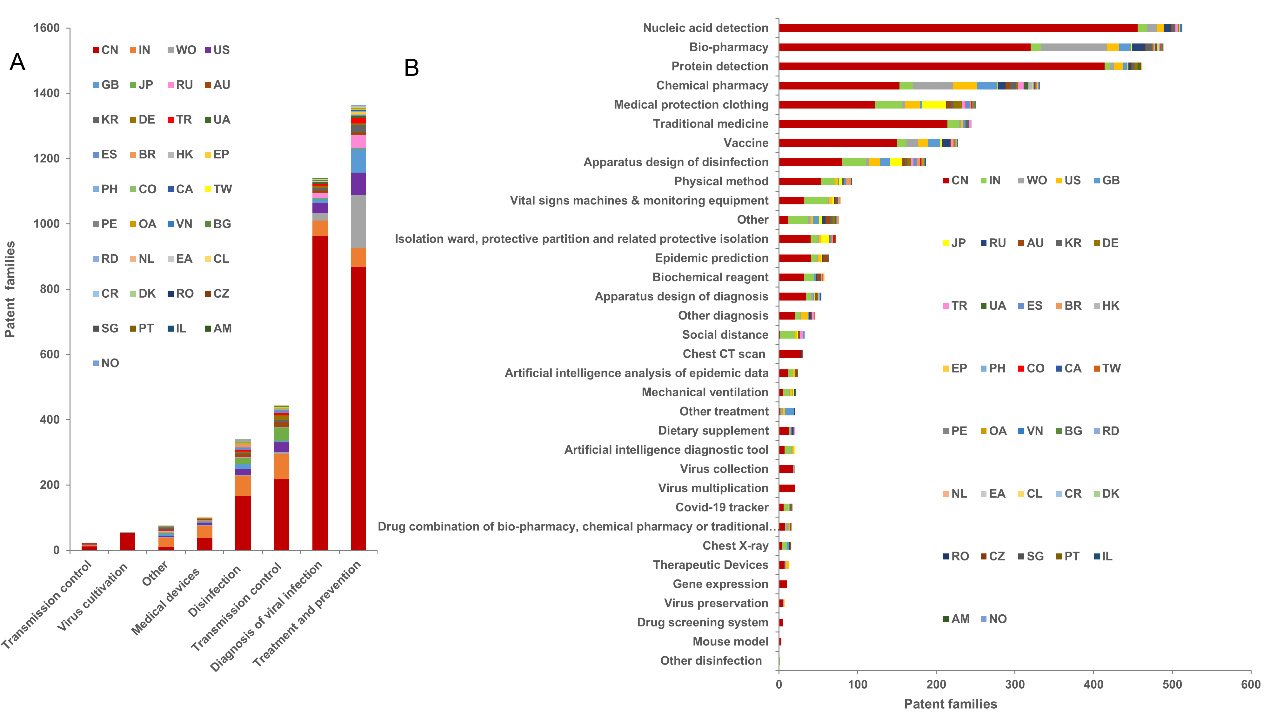


**Figure.5 Number of patent families per country directly related to per technical category. (A)** **Technology groups by regional distribution;(B)** **Technology subcategories by regional distribution.**


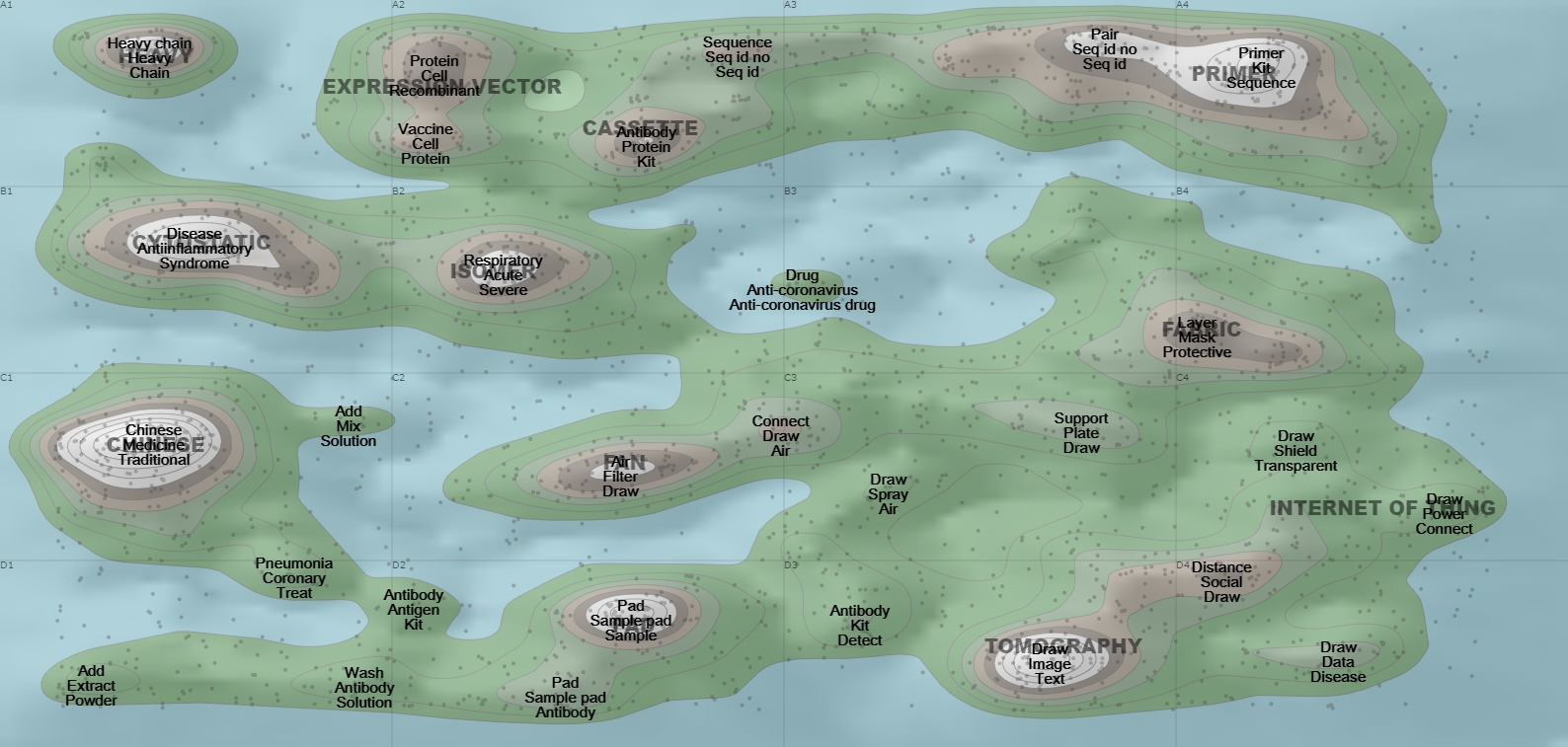


**Figure.6 The landscape by ThemeScape using information included in title abstract and claims of the COVID-19 patents. The patents were delineated and grouped by contour lines showing areas of high (white ‘snow-capped’ peaks) and low patenting activity organized into common themes.**


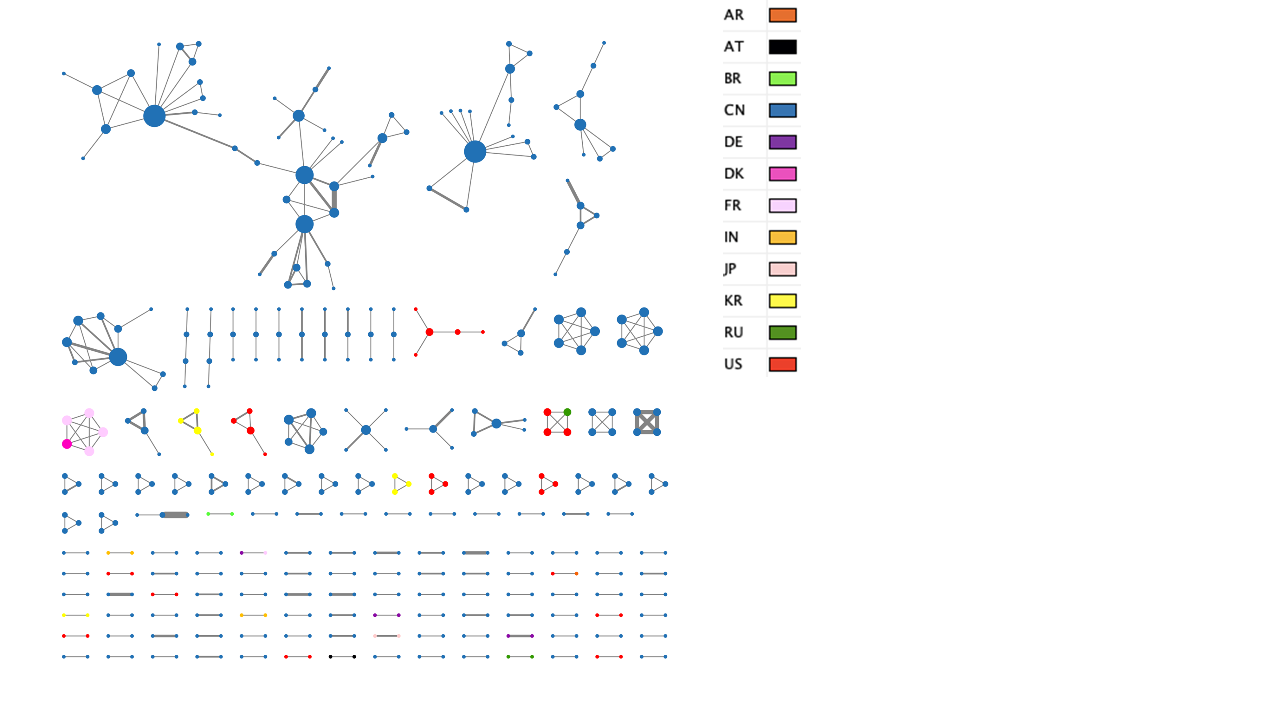


**Figure.7 The information-rich collaboration network among co-assignees of COVID-19 patents. In the network, nodes denote assignees, and edges represent co-assignee relationships. Node size is scaled to the number of patent families owned by the assignee. The thickness of edges represents collaboration frequency.**


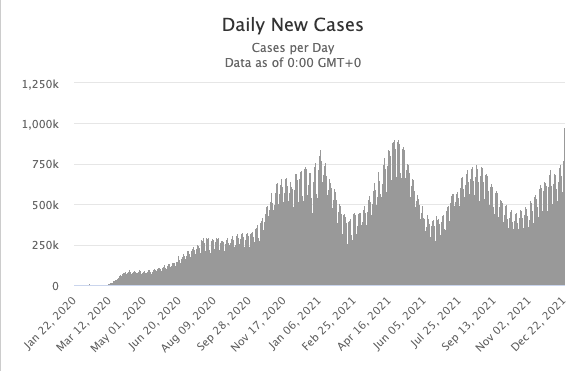


**Figure.8 Daily confirmed COVID-19 cases, World.(**[**https://ourworldindata.org/coronavirus-data**](https://ourworldindata.org/coronavirus-data)**)**

**4. SUPPLEMENTARY MATERIAL TABLES**

**Table 1. Family size of COVID-19 patents by the location of assignees.**

| Location | Average patents family size | No. patent families | No.patent documents |
| --- | --- | --- | --- |
| IL | 1.57 | 7 | 11 |
| JP | 1.33 | 3 | 4 |
| CA | 1.31 | 13 | 17 |
| US | 1.23 | 330 | 405 |
| GB | 1.20 | 15 | 18 |
| IR | 1.20 | 5 | 6 |
| DE | 1.07 | 46 | 49 |
| RU | 1.06 | 67 | 71 |
| CN | 1.04 | 2340 | 2425 |
| IN | 1.02 | 332 | 338 |
| TR | 1.00 | 30 | 30 |
| BR | 1.00 | 17 | 17 |
| CH | 1.00 | 14 | 14 |
| UA | 1.00 | 14 | 14 |
| KR | 0.98 | 46 | 45 |

**Table 2. Country codes of patents by jurisdiction.**

| **Abbreviation** | **Full name** |
| --- | --- |
| CR | Costa Rica |
| DK | Denmark |
| RO | Romania |
| CZ | Czech Republic |
| SG | Singapore |
| PT | Portugal |
| IL | Israel |
| AM | Armenia |
| NO | Norway |
| LU | Luxembourg |
| NL | Netherlands |
| CL | Chile |
| VN | Vietnam |
| BG | Bulgaria |
| EA | Eurasian Patent Organisation |
| OA | African Intellectual Property Organization |
| TW | Chinese Taipei |
| PE | Peru |
| PH | Philippines |
| CO | Colombia |
| CA | Canada |
| EP | European Patent Office |
| HK | Hong Kong (China) |
| BR | Brazil |
| UA | Ukraine |
| ES | Spain |
| TR | Turkey |
| DE | Germany |
| KR | Republic of Korea |
| AU | Australia |
| RU | Russian Federation |
| JP | Japan |
| GB | United Kingdom |
| US | United States of America |
| WO | World Intellectual Property Organisation (WIPO) |
| IN | India |
| CN | China |
| US | United States |
| KR | Korea, Republic Of |
| IR | Iran, Islamic Republic Of |
| CH | Switzerland |
| FR | France |
| BY | Belarus |
| TW | Taiwan |
| SA | Saudi Arabia |
| HK | Hong Kong |
| AT | Austria |
| CM | Cameroon |
| IT | Italy |
| AE | United Arab Emirates |
| VN | Viet Nam |
| DO | Dominican Republic |
| EG | Egypt |
| DZ | Algeria |
| SE | Sweden |
| IQ | Iraq |
| BM | Bermuda |
| KN | Saint Kitts and Nevis |
| PL | Poland |
| NZ | New Zealand |
| FI | Finland |
| AR | Argentina |
| BE | Belgium |
| SH | Saint Helena |
| ER | Eritrea |
| MA | Morocco |
| MY | Malaysia |
| GA | Gabon |
| TO | Tonga |
| NE | Niger |
| IO | British Indian Ocean Territory |
| RE | Reunion |
| KI | Kiribati |
| NU | Niue |
| FO | Faroe Islands |
| ST | Sao Tome and Principe |
| SB | Solomon Islands |
| SO | Somalia |
| SK | Slovakia |
| MK | Macedonia, the Former Yugoslav Republic Of |
| CV | Cape Verde |
| MS | Montserrat |
| TZ | Tanzania, United Republic Of |
| RS | Serbia |
| TC | Turks and Caicos Islands |
| NC | New Caledonia |
| NP | Nepal |
| JM | Jamaica |
| SN | Senegal |
| EM | Office for Harmonization in the Internal Market (Trade Marks and Designs) (OHIM) |
| QA | Qatar |
| ME | Montenegro |
| ZA | South Africa |
| MM | Myanmar |
| RD | Research Disclosures |

**Table 3. Technology of IPC code.**

| IPC | Meaning |
| --- | --- |
| A61P003114 | Antiinfectives for RNA viruses |
| G01N0033569 | Investigating or analysing materials for microorganism |
| C12Q000170 | Measuring or testing processes involving enzymes or microorganisms (measuring or testing apparatus with condition measuring or sensing means); Processes of preparing such compositions, involving virus or bacteriophage. |
| A61P001100 | Drugs for disorders of the respiratory system |
| C12N001511 | Mutation or genetic engineering; DNA or RNA concerning genetic engineering, vectors |
| C12R000193 | Microorganisms |
| A61K0039215 | Medicinal preparations containing antigens or antibodies for Coronaviridae |
| G01N0033558 | Investigating or analysing materials by using diffusion or migration of antigen or antibody |
| G01N0033543 | Investigating or analysing materials with an insoluble carrier for immobilising immunochemicals |
| C07K001610 | Immunoglobulins from RNA viruses, e.g. monoclonal or polyclonal antibodies |
| G01N003368 | Investigating or analysing materials by specific methods not covered by groups involving proteins, peptides or amino acids. |
| A41D001311 | Protective face masks, e.g. for surgical use, or for use in foul atmospheres |
| C12Q00016844 | Measuring or testing processes involving enzymes or microorganisms ; Compositions therefor; Processes of preparing such compositions involving nucleic acids amplification reactions |
| G01N0033577 | Investigating or analysing materials by specific methods involving monoclonal antibodies |
| G01N003358 | Investigating or analysing materials by specific methods involving labelled substances |
| C12Q0001686 | Preparing nucleic acids for analysis, e.g. for polymerase chain reaction [PCR] assay |
| C07K0014165 | Derivatives thereof, Coronaviridae |
| A61K003942 | Medicinal preparations containing viral antigens or antibodies |
| A61P003116 | Antiinfectives, i.e. antibiotics, antiseptics, chemotherapeutics, for influenza or rhinoviruses |
| C12N001513 | Mutation or genetic engineering for immunoglobulins |

**Table 4. The standardization of patent classification.**

| **Case** | **Classification** |
| --- | --- |
| Title | If the classification information is clearly shown in the title of a patent document, we labelled the patent by the classification. For example, patent CN111375055 is entitled “Vaccine composition useful against novel coronavirus (2019-nCoV) and HIV, comprises virus envelope subunit vaccine and glucopyranosyl lipid adjuvant”. We chose the “Vaccine” as the classification. |
| Abstract | If there is no classification information mentioned in the title, we further captured the classification information in the abstract. For example, the abstract of patent CN111647055 is “The invention belongs to the technical field of immunization, and particularly relates to a recombinant antigen for novel coronavirus detection and preparation and application thereof.” We chose the ”Diagnosis of viral infection“ as the classification. |
| Claims | If there is no classification information both in title and in abstract, we continued to check patent claims. In most cases, by reading the title and abstract, we can divide the patents into general categories. If we can't determine the subcategory by reading the title and summary, we need to refer to patents claims to classify the subcategories under the general category. Make decisions based on the level of priority claim. For example, patent US2020281972 claims that “Claim 1. A method of treating at least one condition caused by a coronavirus or an influenza virus, the method comprising administering a composition comprising copper ions to a subject in need thereof.” We recognized that the patent covers "Chemical pharmacy" under “treatment and prevention”. |
| Full-text | When none of the above ways can determine the category, we will read the full text. |
